# The molecular insights into protein adsorption on hematite surface disclosed by in-situ ATR-FTIR/2D-COS study

**DOI:** 10.1038/s41598-020-70201-z

**Published:** 2020-08-10

**Authors:** Matheus Sampaio C. Barreto, Evert J. Elzinga, Luís Reynaldo F. Alleoni

**Affiliations:** 1 AgroBiosciences Division, Mohammed VI Polytechnic University (UM6P), Lot 660, Hay Moulay Rachid, 43150 Benguerir, Morocco; 2 Department of Soil Science, Luiz de Queiroz College of Agriculture (ESALQ), University of São Paulo (USP), Piracicaba, SP Brazil; 3 Department of Earth & Environmental Sciences, Rutgers University, Newark, NJ USA

**Keywords:** Biogeochemistry, Environmental sciences

## Abstract

Proteins are of ubiquitous interest in the Life Sciences but are of interest in the Geosciences as well because of the significant role these compounds play in the biogeochemical cycling of trace and nutrient elements. Structural changes resulting from the adsorption of proteins onto mineral surfaces may alter protein biological function and other environmental interactions. Iron oxides are major sinks of a range of environmental elements including organic compounds. In this study, the adsorption of the broadly studied model protein BSA onto the hematite mineral surface was characterized as a function of pH, ionic strength, and BSA concentration using in-situ Attenuated Total Reflection Fourier Transform Infrared (ATR-FTIR) spectroscopy. BSA lost the α-helix and gain β-sheets in the secondary structure during adsorption on hematite. BSA adsorption was maximum at pH 5, a value close to the BSA isoelectric point (~ pH 5), and lower at pH 4 and pH 7. Increasing ionic strength decreased to total BSA adsorption. Two-dimensional correlation spectroscopy analysis of the ATR-FTIR spectra revealed that higher initial BSA concentration and the consequent higher BSA surface loading enhanced BSA adsorption by protein–protein interaction, which less ordered structures changes into more compact forms decrease, hence compacting the structural arrangement and could promoting multilayers/aggregation formation on the mineral surface. The activity of enzymes following adsorption on mineral surfaces requires further study.

## Introduction

Proteins are of ubiquitous interest in the Life Sciences. Their behavior is relevant to several scientific fields including the biochemistry of early Life^1,2^, pathogen-host interactions^3,4^, food chemistry^5^, human health^6–10^, industrial reactions^11–13^, drugs development^14–16^, and microbial biofilm formation^17–22^. They are transferred into the environment during cellular lysis or biological activity^23^.

Interest in the Geosciences regarding protein behavior is the result of its role in the biogeochemical cycling of trace and nutrient elements. Enzymes are of paramount influence to the plant-availability of nutrients such as N^24–26^, P^25,27^, and S^28^ , and to C mineralization^24,26,29^. Many studies provide evidence for the importance of microbial-derived soil organic matter in soils^30,31^, with the proteinaceous compounds being major contributors to soil organic matter persistence and accumulation. N-containing compounds can adsorb directly onto mineral surfaces which decreases their degradation by other catalytic enzymes^31,32^.

Proteins in soil environments, as enzymes, are subject to adsorption onto the surfaces of the mineral fraction. This should affect the protein's biological functions^33–35^, as its functionality is determined by protein chemical and physical properties, which in turn are determined by the interactions between its amino acid residues in three dimensional space^36^. The adsorption of proteins at solid surfaces has been characterized as “a common but very complicated phenomenon”^37^. Protein adsorption is driven by multiple forces, including cation exchange, van der Waals and electrostatic interactions, hydrogen bonding, and hydrophobic interactions. The structural arrangement of the protein is determined by these interactions, and is termed the secondary structure, which is of great interest because it affects protein functioning.

Secondary structure can be determined by IR spectroscopy, because structural arrangements in the protein chain are associated with specific vibrational bands^38–46^. Besides IR, other techniques used to determine structure include X-ray crystallography^47^ and UV-circular dichroism^46,48–50^.

Environmental variables such as the nature of the adsorbent surface (e.g. hydrophobicity) and protein properties (e.g. molecular weight, amino-acids composition and sequence), medium pH, ionic strength, others ions/compounds in to the system are understood to influence these protein adsorption forces, which guides to a complex organo-mineral system with simultaneously kinetic, structural, and thermodynamic controls. An extensive discussion on this topic is presented in previous studies^36,46,51–58^.

Iron oxides are abundant mineral constituents of soils, where they occur as the products of weathering and bacterial processes, and act as a source of the nutrient iron^59^. During rock weathering, Fe is released and subsequently precipitated as ferric oxides and hydroxides^60^. Iron oxides are major sinks to several environmental compounds including organic^61^ and inorganic pollutants^62^ and control the availability of plant nutrients in soils^63^.

Hematite (α-Fe_2_O_3_) is the most thermodynamically stable and often the most abundant iron oxide in soils and sediments among a number of other polymorphs of iron oxides and oxyhydroxides^59,60^, with common occurrence in weathered tropical soil^64,65^. This mineral has an “corundum crystal” type structure, with layers of oxygen atoms distorted hexagonally in a close-packed configuration, divided by a double layer of Fe^3+^, which has a 2/3 occupancy of the octahedral sites in sequence of –(Fe–O_3_–Fe)– alongside the *c* axis^66^. The mineral surface has an elevated free energy of formation and surface enthalpy, which promotes strong water adsorption and interaction with ions^59,66^. Although it is less common in soils than goethite (α-FeOOH), water adsorption experiments have shown that hydrated hematite surfaces behave thermodynamically as goethite surfaces^66^.

Bovine serum albumin (BSA) is suitable for adsorption studies because of its high stability, availability, and purity, and its low cost^44,67,68^. This protein is a widely used in studies of protein-surfaces interactions^69^. BSA and human serum albumins exhibit similar folding, a well-recognized primary structure, and they have been associated with the binding of many types of small molecules^6,58,67^. Once a protein is associated with a surface, processes such as protein reorientation can induce conformational changes accompanied by protein unfolding, lateral protein–protein interactions, and desorption^36,46,56,68^. Structural changes of an adsorbed protein may alter protein biological function and other environmental interactions^11,13^. Because of its ability to observe structural changes in adsorbed protein by Amide I band analysis^38,39,46^, Fourier Transform Infrared (FTIR) spectroscopy is a useful tool for determining the secondary structure of proteins. *In-situ* Attenuated Total Reflectance FTIR (ATR-FTIR) spectroscopy is a technique that permits the study of interfacial processes at the molecular scale of conformational change of proteins upon adsorbed in real time^70^. The technique has been successfully applied to study the interaction of Fe-oxide with inorganic oxyanions such as phosphate^71^ and recently for protein adsorption on clay material^44^ and hematite^46^.

The use of ATR-FTIR in combination with data analysis by two-dimensional correlation spectroscopy (2D-COS) is well suited for the interpretations of spectroscopic signals of chemical processes that are develop in response to an external disturbance, such as variations in pH, concentration, and time. 2D-COS can be used to determine the sequential appearance of features resulting from such changes. Recently, 2D-COS was used to analyze protein adsorption^44,46^ and denaturation processes^72,73^, highlighting the potential of 2D-COS application to protein structural analysis as a function of temperature^74^, concentration^44^, pH^75^, and mineral size^46^. Therefore, the aim of this study was to provide new insights into the adsorption of BSA onto the hematite mineral surface as a function of pH, ionic strength, and BSA concentration. It is hypothesized that these environmental conditions effects the amount of protein adsorption and it secondary conformation.

## Materials and methods

### Materials

**Figure 1:**
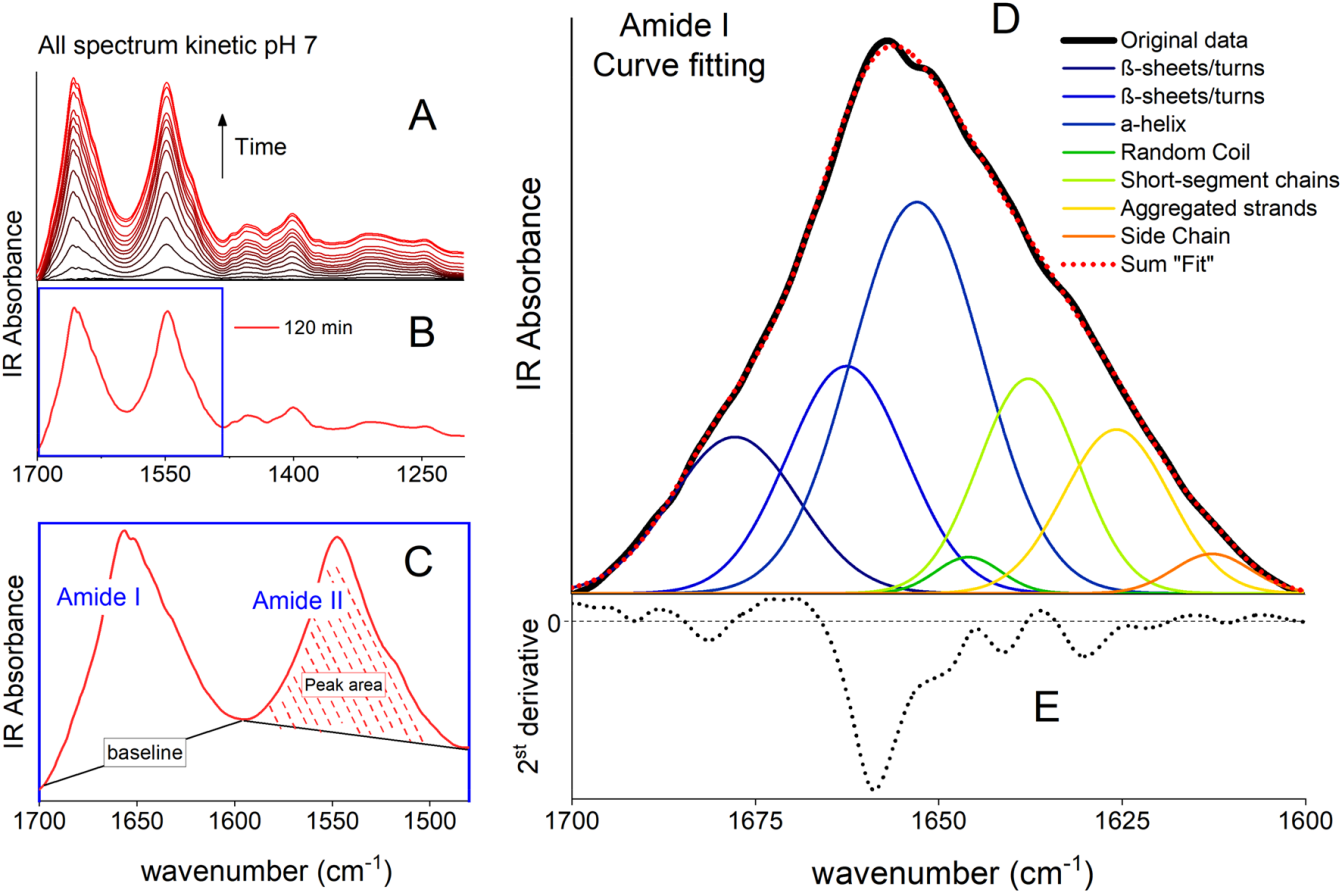
Process of kinetic data analysis: (**A**) Raw data of BSA adsorption kinetic experiment collected between 1 min (bottom black line) and 120 min (top red line) of reaction time. (**B**) Individual spectrum of the 120 min spectrum. (**C**) Double baseline fit of the Amide I and II band in the 120 min spectrum. The Amide II band area was integrated and is presented in Fig. 4. (**D**) The Amide I band was deconvoluted into Gaussian curves at peak position initially determined by the 2nd derivative spectrum of the original data, which is shown in panel (E).

The hematite sorbent used for the experiments was synthesized based on a published procedure^76^ and has been used previously^77^; a detailed description of the material is provided in^78^. X-ray diffraction confirmed that the material does not contain crystalline phases other than hematite. ATR-FTIR analysis (Fig. 1SM) shows the absence of IR peaks overlapping with the amide I band region of BSA protein. The N_2_-BET specific surface area and the zero point of charge (ZPC) were 24 m^2^ g^−1^ and 9.3, respectively.

The protein used here was the bovine serum albumin (BSA) (fraction V, purchased from Sigma-Aldrich), which has an isoelectric point at pH 4.9–5.1^44,79^ and a molecular weight of ≈ 66.5 kDa. All chemical reagents were of analytical grade, and Milli-Q water (18.2 MΩ cm) was used throughout the experiments.

### In-situ ATR-FTIR experiment

The experimental IR setup is very similar to that used by Refs.^77,80,81^. Data acquisition was performed with a IR spectrometer (PERKIN–ELMER, model SPECTRUM 100) equipped with a purge gas generator (BALSTON–PARKER) and a liquid N_2_-cooled mercury–cadmium–telluride detector. ATR-FTIR flow-cell containing a horizontal ZnSe crystal (10 internal reflections; 45° angle of incidence). The crystal was installed in the flow cell inside the IR spectrometer and connected by Teflon tubes to a vessel containing 100 mL of 10 mM NaCl electrolyte which was magnetically stirred and set to the desired pH. A peristaltic pump was used to flow solute from the reaction vessel through the cell at a rate of 2 mL min^–1^. All experiments were carried out at 22 ± 2 °C.

Aqueous BSA spectra were collected using ATR-FTIR flow-cell crystal without hematite coating. The flow-cell was equilibrated (20 min) with 10 mM NaCl and an ATR-FTIR spectrum was collected as a background spectrum (100 scans). Then, BSA was added to the vessel to reach a 0.15 mM of BSA concentration (≈10 g L^–1^). The initial spectrum (100 scans) was collected at pH 8.7. pH was lowered stepwise to pH 4 by the progressive addition of small amounts of 0.1 M HCl. At each pH step, a new spectrum was collected to characterize the pH dependence of the speciation of aqueous BSA.

The sorption experiments were conducted with the ATR-FTIR flow-cell coated with hematite. The mineral film was made by applying 125 µL of a 5 g L^–1^ hematite suspension to ~ 1 ml water, and spreading the resulting suspension evenly across the ATR crystal surface. Drying at room temperature overnight produced a stable and homogeneous deposit. A new hematite film was prepared for each experiment. After the experiments, the hematite deposit was removed from the flow cell crystal by gentle scrubbing with a kimwipe and detergent using abundant water, followed by extensive rinsing with DDI water. The other components of the flow setup (tubes and vessel) were cleaned with detergent solution followed by extensive rinsing with DDI water.

The hematite thin-film deposited on ZnSe crystal was initially equilibrated with the background solution for ~ 2 h, and then a background spectrum containing the absorbance of: the the hematite + ZnSe crystal + the background electrolyte, 100 scans were co-added at a resolution of 4 cm^–1^. Posteriorly, the aqueous BSA was spiked into the vessel to start the BSA adsorption experiments. The spectra’s of adsorbed BSA were corrected by background spectrum collected previously. The solution pH in the vessel was monitored throughout the experiment and readjusted or changed as necessary by addition of small aliquots of 0.1 M NaOH or 0.1 M HCl.

Three main types of adsorption experiments were carried out, each aimed at characterizing a specific aspect of BSA interaction with the hematite surface. First we determined the effects of surface loading on the BSA conformation on the hematite surface by varying the BSA solution concentration at pH 5, as in previous work^44^. After pre-equilibration (~ 2 h) of the hematite deposit with background electrolyte (10 mM NaCl), BSA was added to reach 0.2 µM, and was then allowed to achieve adsorption equilibrium, which required ~ 20 min. After collecting the spectrum of adsorbed BSA, the solution concentration was raised in a step-wise fashion up to [BSA] = 1.5 µM. At each BSA concentration, aqueous BSA was allowed to reach adsorption equilibrium (typically requiring ~ 20 min), and the ATR-FTIR spectrum of adsorbed BSA was collected before raising the concentration to the next level.

In a second set of experiments, we tested the effects of ionic strength on BSA conformation and loading on the hematite surface. After pre-equilibrating the hematite deposit at pH 5 and 1 mM NaCl, aqueous BSA was added at a concentration of 1 µM, and allowed to achieve adsorption equilibrium (20 min). Next, NaCl salt was added to the vessel in a stepwise fashion to reach NaCl concentrations in the range [NaCl] = 1–100 mM. At each ionic strength, BSA adsorption was allowed to reach equilibrium with the hematite deposit before the spectrum of adsorbed BSA was collected and the ionic strength was raised to the next level.

**Figure 2:**
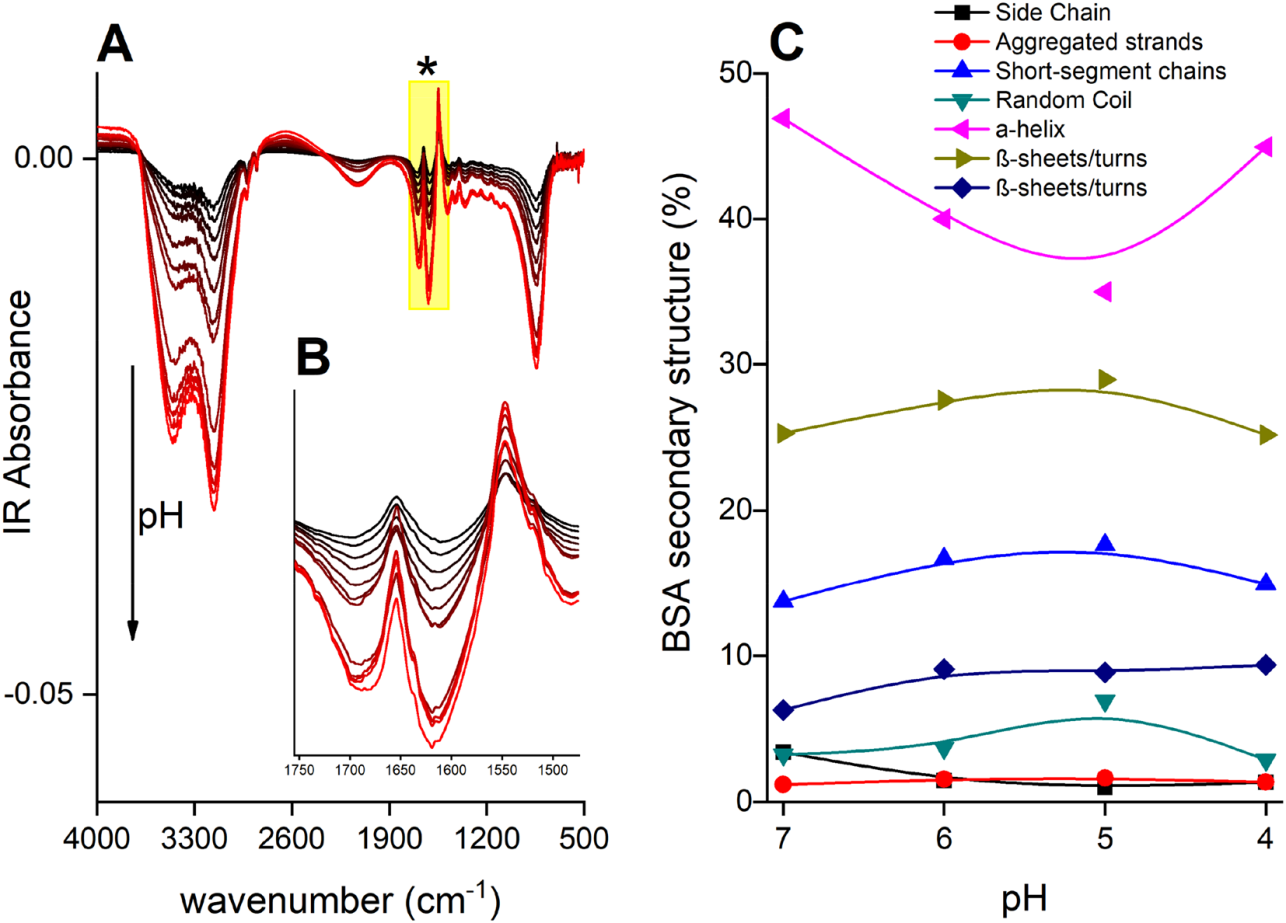
(**A**) Raw IR spectra of aqueous BSA (15 mM L^–1^) collected over the pH range from 8.7 (top black line) to 4 (bottom red line) in 10 mM L^–1^ NaCl. The amide bands are located in the yellow-highlighted spectral region indicated by asterisk (**B**) Zoom-in of the spectral region containing the Amide I and II bands. Water IR absorption (υ_2_ H_2_O) at 1644 cm^–1^ overlaps with the Amide I band (1700–1600 cm^–1^) but not with amide II. (**C**) Relative band intensities as a function of pH indicate Secondary structure proportion of free aqueous-BSA at pH 7, 6, 5, and 4. See the corresponding structural assignments in relation to wavenumber peak in the Table 1.

The final set of experiments aimed to characterize the adsorption and desorption kinetics and dynamic changes in the BSA conformation at pH 4, 5, 6, and 7. At each pH, the hematite deposit was pre-equilibrated with background electrolyte and then BSA was added at a concentration of 1 µM L^–1^ (66.5 mg L^–1^), and its adsorption onto the hematite deposit was tracked by regular collection of spectra between 1 and 120 min after the BSA addition (Fig. 2SM). The kinetic spectra were recorded as the average of 20 scans at a 4 cm^–1^ resolution, requiring ~ 18 s per spectrum. At the end of each experiment, we induced BSA desorption by switching to a new solution consisting of background electrolyte (10 mM NaCl) of the same pH, but without BSA. The desorption solute was collected as waste after passing through the flow-cell. After 30 min, the spectrum of BSA that remained adsorbed on the hematite film was collected.

### Data processing

#### Quantitative analysis

The method used for data processing is illustrated in Fig. 1. A semi-quantitative estimate of BSA surface loading was obtained by integration of the area under the Amide II vibrational band (1600–1480 cm^−1^) (Fig. 1C). This band was chosen for these analyses because, compared to the amide I band vibration (1700–1600 cm^−1^), it is less affected by interference of the water bending mode at ~ 1645 cm^−1^ and less affected by conformational changes related to the adsorption process^39,44^. The baseline was fitted as a straight line between 1600 and 1480 cm^–1^ and the area under the peak determined by integration using the MagicPlot ver. 2.7.2 software (MAGIC PLOT SYSTEMS, LLC, Saint Petersburg, Russia). Data processing of the kinetic and isotherm IR data process was carried with the Origin 2018 software package (ORIGINLAB CORPORATION, Northampton, Massachusetts, USA).

#### Protein conformational structure

The IR data of adsorbed BSA were analyzed with quantitative Gaussian peak fitting to determine secondary structure^39^. The method is illustrated in Fig. 1. Briefly, we used fit a linear baseline across the Amide I band (1700 − 1600 cm^–1^), and then the second derivative spectrum was determined to find the position and number of structural component bands for peak fitting. Once the component positions were identified and the baseline established, Gaussian peaks were added to the Amide I band according to the position suggested by the second derivative spectrum (Fig. 1D). This procedure was used for the aqueous spectra and for the kinetic spectra obtained after 120 min for each pH.

In the peak deconvolution process, the initial peak position of Gaussians curves was determined by the second derivative (Fig. 1E). The width was equal for all curves whereas the heights were manually adjusted to obtain a “visual” good fitting result. Then, using peak positions fixed, we applied the Levenberg−Marquardt algorithm to minimize the residual sum square of the fitted and experimental spectra. To end with, the fitting was repeated using width equal for all curves and allowing the peak positions to shift. This step-by-step fitting process allows a comprehensive first estimative for curve parameters, which is crucial for success of iterative process used by algorithm^82^.

The peak positions reach a shift among aqueous-BSA and adsorbed BSA about ± 5 cm^–1^, regardless of pH value; and within ± 8 cm^–1^ of the position determined by the second derivative. The coefficient of determination (R^2^) was ≥ 0.996 for deconvolution fits. The pertinent structures observable in the Amide I region of IR spectra by some authors are summarized in Table 1; these include α-helix, β-sheet, and random coil structures, among others.

**Table 1:** Vibrational frequencies (cm^–1^) of the absorption bands associated with secondary structure conformation of BSA in the Amide I region used for curve fitting.

Secondary structure assignment	Vibrational frequency range (cm^–1^)				
	Ref.^89^	Ref.^42^^a^	Ref.^44^	Ref.^46^	Peak center found in this work
β-Sheets/turns	1685–1663	1690–1680	1690–1660	1690–1660	1676
β-Sheets/turns		1670–1662			1662
α-Helix	1655–1650	1655–1650	1660–1650	1655–1650	1652
Random coil	1648–1644	1645–1642	1648–1644	1648–1644	1644
Extended chains/β-sheets/short-segment chains connecting the α-helical segment^2^	–	1638–1632	1640–1630	–	1637
Extended chains/β-sheets, Aggregated strands	1639–1621	1620–1610	1630–1620	1640–1620	1628
Side chain moieties	1616–1600	–	1620–1600		1612

^a^The authors used D_2_O solution, Refs.^41,42^.

#### 2D-COS analyses

The Amide I bands (1700 − 1600 cm^–1^) obtained from the analyses described in “Protein conformational structure”. were used for the 2D correlation analysis. Before analysis, the peaks spectrums were normalized by multiplicative scatter correction. This step is crucial in order to assess the changes that accompany the external perturbation factor of interest, which occur concomitantly with changes in the extent of surface adsorption^44,83,84^. We evaluated the effects of BSA and NaCl concentration at pH 5, as well as the effect of time in the adsorption kinetics at pH 4, 5, 6 and 7. The 2D-COS analyses were carried using 2DShige version 1.3 software (Shigeaki Morita, Kwansei-Gakuin University, 2004–2005.). The synchronous and asynchronous maps obtained from 2D-COS analysis were plotted using Origin 2018. The “reaction order” interpretation followed Noda rules^85,86^. A detailed description of the mathematical approach of 2D-COS analyses is provided elsewhere^85,86^.

## Results

### Aqueous BSA

Acquisition of ATR-FTIR data of proteins in aqueous solution is not straightforward due to a number of experimental challenges. The first is that relatively high concentrations are needed to obtain spectra of reasonable quality^39,87^. This is due to the low affinity of protein for the ATR crystal surface, as the result of electrostatic repulsion between BSA and ZnSe and the weakly hydrophobic nature of the ZnSe surface^88^. In the pH range 5–8.4, both the ZnSe surface (isoelectric point = 4.9^89^) and dissolved BSA (isoelectric point = 4.9–5.1) are negatively charged, resulting in electrostatic repulsion.

Protein accumulation in the near-surface region of the ZnSe crystal probed by the IR beam may be driven by hydrophobic effects at these pH values. The hydrophobic effect could be suggested by preferential desorption of interfacial “ice-like” water (3400 cm^–1^ peak; Fig. 2A), which has a more oriented structure than bulk water (3200 cm^–1^)^51^. At lower pH, (red lines in Fig. 2A), the charge densities of dissolved BSA protein and the ZnSe crystal surface decreased, thereby reducing both protein–protein and protein-surface repulsion. This resulted in increased BSA surface loadings (Fig. 2A), although the overall intensities remain relatively low even at high BSA solution concentration.

A further challenge for in-situ ATR-FTIR analysis of aqueous protein is the interference of water bands in the IR region. As seen in Fig. 2B, water has a strong IR absorbance around 1640 cm^–1^ (H–O–H bending), which overlaps with the Amide I mode of BSA (1700 cm^–1^ and 1600 cm^–1^). Despite these experimental challenges, we succeeded in acquiring BSA spectra over the pH range of interest here that could be used to characterize the BSA secondary structure because of the high signal/noise provided by the ATR-FTIR setup, as well previous studies^6,44,46,48,90,91^.

In aqueous media, interactions of the hydrophilic side chains with water are maximized through hydrogen bonding, while interactions of hydrophobic side groups are minimized. The protein secondary structure of aqueous free-BSA had the α-helix conformation as the prevalent form (Fig. 2C), with estimated contributions of 47%, 40%, 35%, 45% at pH 7.0, 6.0, 5.0, and 4.0 respectively. We found similar results of α-helix content in the total secondary structure of aqueous BSA, similar to others studies^40–45^. Literature values for native BSA secondary structure showed a variation around ± 10% among secondary structures distribution, depending on the experimental setup, the spectroscopic technique used for characterization, and experimental conditions including BSA concentration and electrolyte type and concentration.

### BSA adsorption onto hematite: initial protein concentration effects

**Figure 3:**
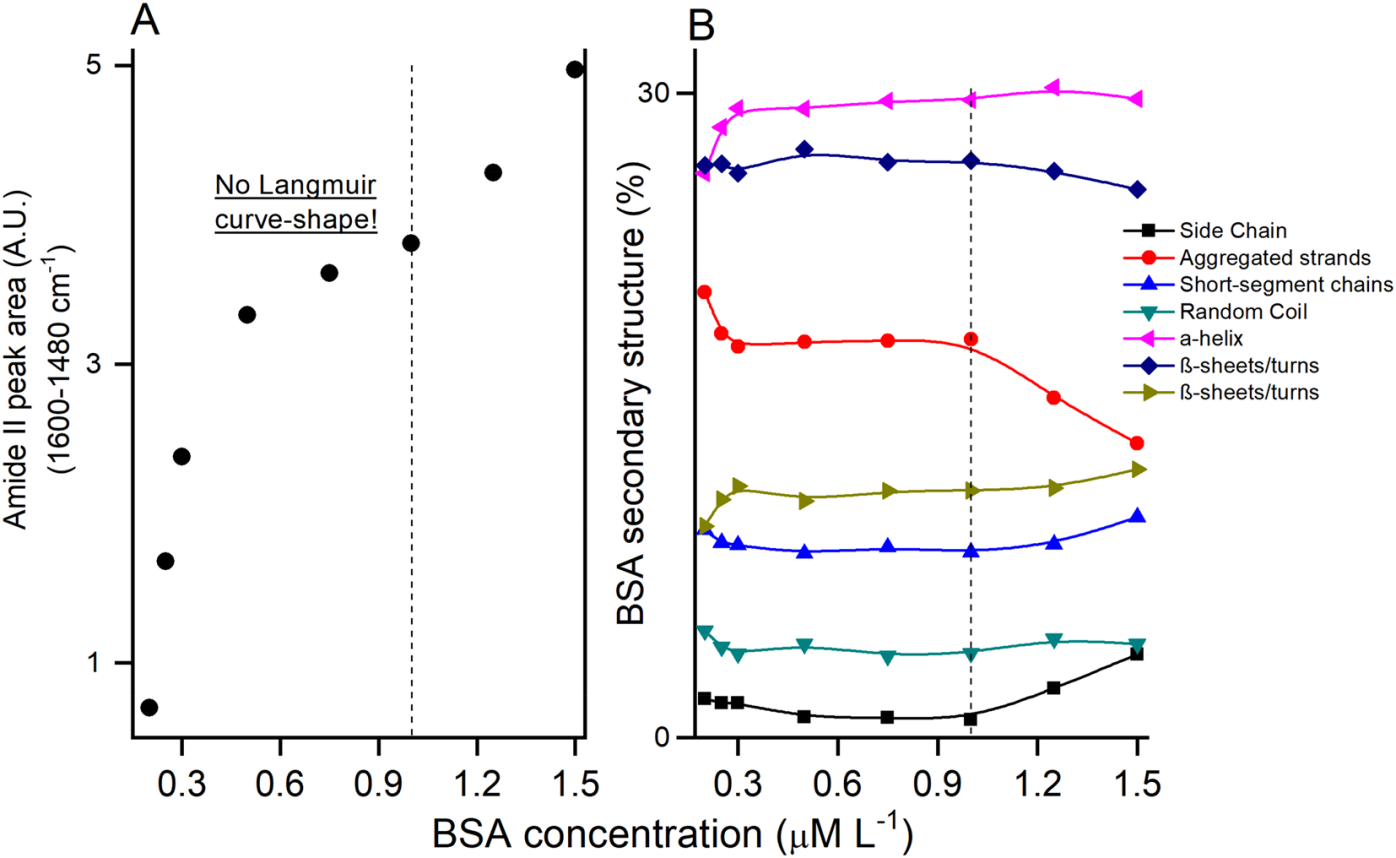
(**A**) Adsorption for BSA adsorption onto hematite over BSA concentration at pH = 5 and [NaCl] = 10 mM L^–1^. (**B**) Secondary structure proportion of adsorbed BSA on hematite. See the corresponding structural form in relation to wavenumber peak in Table 1. Dotted line represents the BSA concentration used to kinetic experiment.

Isotherm data of BSA adsorption on hematite were derived from the Amide II areas in the IR spectra of adsorbed BSA at aqueous BSA concentrations that ranged from 0.2 to 1.5 µM (Fig. 3). The isotherm data do not conform to the Langmuir model, in that no surface saturation was observed (Fig. 3A). Our findings differ from the Langmuir-type adsorption of BSA in batch experiment with oxides^92–94^ and clay materials^95–97^, as well as with the results of and *in-situ* ATR-FTIR study of BSA adsorption on montmorillonite^44^ performed with an experimental setup similar to ours. The results instead suggest an S-shaped isotherm, which represents unrestricted monolayer-multilayer adsorption^98^. The lack of conformity of the isotherm data to the Langmuir model is consistent with a recent study arguing that Langmuir fitting of protein adsorption isotherms is typically inappropriate^99,100^.

IR absorbance values of adsorbed BSA in our in-situ ATR-FTIR experiments reached ~ 0.11 absorbance units at a BSA solution concentration of 1 µM (Fig. 1SM). This is 10 × higher than the IR signal of adsorbed BSA on montmorillonite at a BSA concentration of 3 µM observed in a flow cell system very similar to ours^44^. Clearly, differences in experimental setup likely have contributed to this difference. However, another likely contributing factor is a difference in reactivity of the two sorbents towards BSA. Montmorillonite clay has a negative surface charge which is expected to promote electrostatic repulsion with BSA at the pH values used here. Hematite, on the other hand, has a positive charge at the experimental pH values, which would promote adsorption through electrostatic attraction. Further studies are needed to directly compare adsorption affinities of Fe-oxide and clay sorbents towards BSA and other proteins.

Comparison of the IR spectra of aqueous and adsorbed BSA showed that the secondary structure of adsorbed BSA has changed considerably compared to that of aqueous BSA (Fig. 3B). There is a loss of α-helix (35–30%, 1652 cm^–1^) and short-segment chains connecting the α-helical segment (17–8%, 1637 cm^–1^), concomitant with a gain of aggregated strands (2–17%, 1628 cm^–1^). These changes are consistent with an unfolding process of the protein upon adsorption^43,45^. Similar un-folding process after protein adsorption were observed in previous studies^40,101^.

Nevertheless, the in-situ ATR-FTIR spectra of adsorbed BSA after [BSA] > 1 µM exhibits a decreasing in β-sheets/turns (1662 cm^–1^), and concomitant increments of short-segment connecting α-helix (1637 cm^–1^) and side chain moieties (1612 cm^–1^) (Fig. 3), which confirms the concentration-dependent reaction^102^. This secondary structure shift suggests a higher exposition of hydrophilic groups of side chain and protection of hydrophobic core, similar to the aggregation process^43^. Previous work highlighted the influence of initial protein concentration on adsorption kinetics and extents of BSA on montmorillonite that stressed a more compact and aggregated adsorbed-BSA^44^; and on hematite, which showed an increment at α-helix structure, especially when there are higher BSA concentration^46^.

The 2D-COS analyses of the effects of BSA concentration (Fig. 3SM and Table 2SM) point to a sequence of structural changes in the conformation of BSA upon adsorption at the hematite surface. The arrangement following BSA concentration may be interpreted as^103^: 1620 cm^–1^ (side chain) → 1630 cm^–1^ (aggregated strands) → 1656 cm^–1^ (α-helix). The increment of BSA concentration implies for adsorbed BSA unfolds first into side chains, followed by a loss of aggregated strands and a following adaptation to α-helix. Overall, this corresponds to a conversion of relatively disordered structures into more compact α-helices forms. The increment of α-helix fraction of BSA adsorption at higher protein concentration was observed previously^44^. Hence, we tentatively assign this to surface clustering and aggregation of adsorbed protein molecules, possibly leading to multilayer adsorption^104^, as suggested by the isotherm data (Fig. 3).

By Small-Angle X-Ray scattering technique, the authors^105^ showed the coexistence of monomers and dimers at BSA aqueous concentration (50 mg/mL) and pH 5.4, near the BSA isoelectric point, which the high concentration could be reached close to the surface, as the hematite. These evidences summed with the shape-S adsorption isotherm, instead of Langmuir shape, could suggest a BSA multilayer adsorption on hematite at higher BSA concentration. It is a surface specific phenomenon and requires further study. Based on these isotherm measurements, the remainders of the IR experiments were conducted with a BSA aqueous concentration of 1 µM, because at this concentration we reach maximum BSA adsorption without the strong effects of BSA clustering and aggregation.

### BSA adsorption onto hematite: ionic strength effects

**Figure 4:**
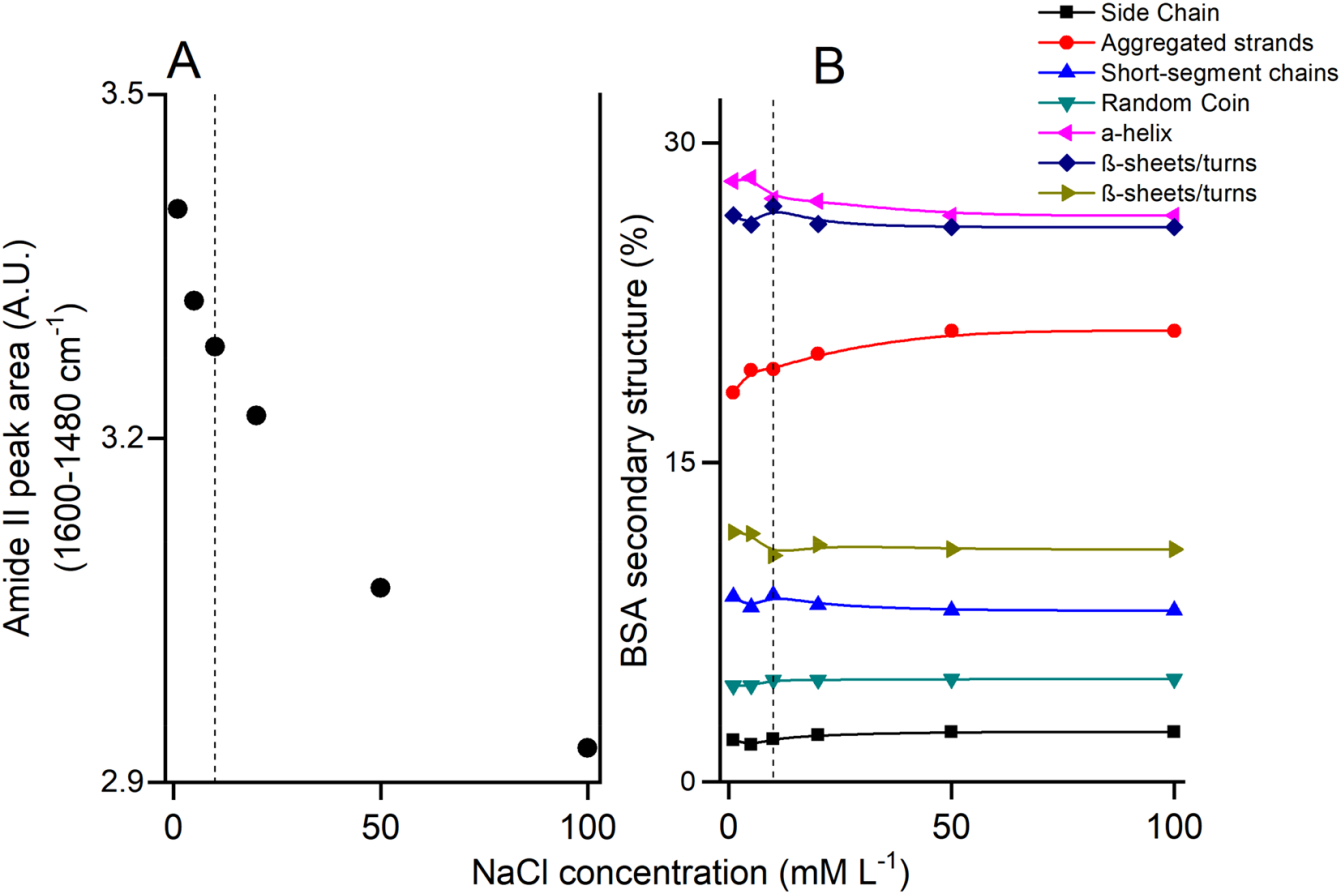
(**A**) Adsorption for BSA adsorption onto hematite over NaCl concentration at pH = 5 and [BSA] = 1 µM L^–1^. (**B**) Secondary structure proportion of adsorbed BSA on hematite. See the corresponding structural form in relation to wavenumber peak in Table 1. Dotted line represents the BSA concentration used to kinetic experiment.

The influence of NaCl concentration in the background electrolyte on the Amide I band of adsorbed BSA is illustrated in Fig. 4. An increase in NaCl concentration 1 to 100 mM results in a decrease of ~ 14% of the amide I band intensity (Fig. 4A), but does not lead to any distinct changes in the secondary structure of adsorbed BSA (Fig. 4B). We attribute the ionic strength effect to decreasing electrostatic attraction between the BSA molecules and the hematite surface as the result of increased charge screening^106^, promoting adsorption of BSA molecules adhered by electrostatic forces or as multilayers structures^99,104^. We speculate that the increase of ionic strength could shield the electrostatic sites on hematite surface, which decrease the electrostatic adsorption between BSA and mineral surface. The 2D-COS results (Fig. 3SM and Table 2SM) suggest the following sequence of structural changes: 1625 cm^–1^ (aggregated strands) → 1615 cm^–1^ (side chain). Overall, this corresponds to a transformation between two less ordered structures.

Ionic strength effects are likely to be dependent on the background electrolyte, in particular the cations, which serve as counter-ions to negatively charged BSA protein. The sodium cations used here do not interact strongly with dissolved anionic macromolecules in solution or at mineral surfaces. However, other common cations such as Ca^2+^ are more reactive, and this may lead to different ionic strength effects than observed here. Interactions between Ca^2+^ and organic molecules in soil systems are well known^107^, and Ca^2+^ may promotes protein aggregation by bridging negatively charged proteins^108^, which may lead to increased protein adsorption with increasing Ca^2+^ solution concentrations. Further study is needed to evaluate the impacts of Ca^2+^ and other common cations (e.g. Mg^2+^, K^+^) on protein interactions with minerals surfaces.

### BSA adsorption kinetics: pH dependency

**Figure 5:**
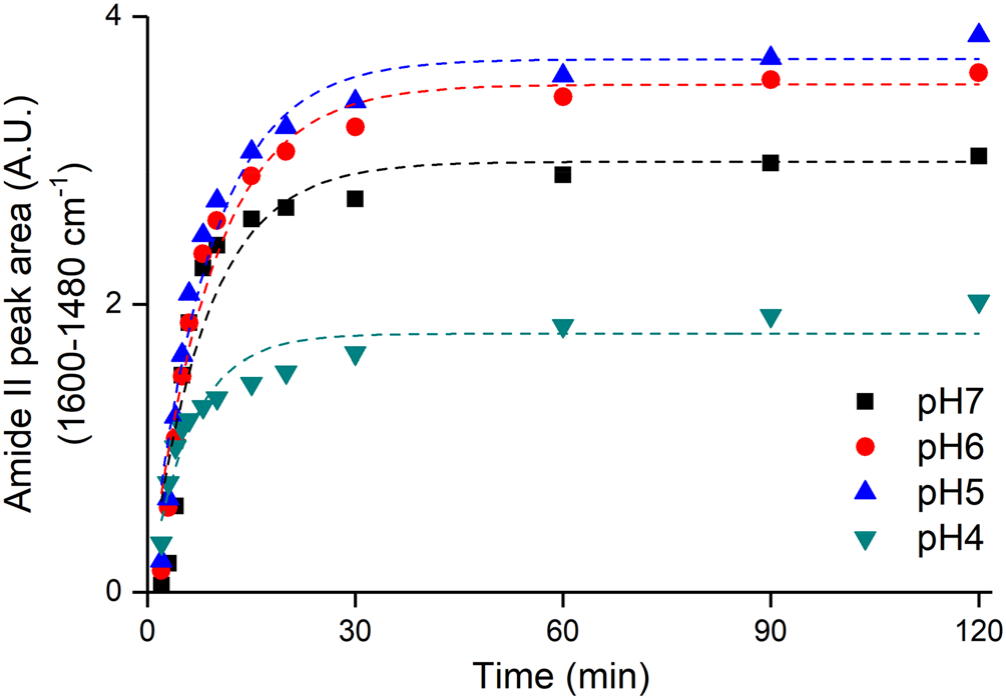
Amide II area of adsorbed-BSA over the time. Pseud-first order kinetic models for each pH are drawer by dashed lines of corresponding color. The [BSA] = 1 µM L^–1^ in electrolytic background (ionic strength) as [NaCl] = 10 mM L^–1^. The equation parameters are listed in Table 2.

Figure 5 displays the semi-quantitative kinetics of the BSA adsorption process as determined from changes in the Amide II area of adsorbed BSA, which are plotted as a function of time intervals for various pH values. These kinetic results were successfully modeled with a pseudo-first order rate model, which yielded higher r^2^ and lower reduced χ^2^ values (Table 2) than other model descriptions (Table 1SM).

**Table 2:** Pseudo-first order kinetic parameters for BSA adsorption and desorbed fraction for each pH value.

pH	AmideII_max_ [peak area (A.U.^2^)]	*K* (1/min)	R^2^	Reduced χ^2^	Desorption (%)
7	2.99	0.119	0.88	0.14	1
6	3.53	0.109	0.95	0.06	5
5	3.70	0.114	0.96	0.07	10
4	1.79	0.164	0.91	0.02	3

BSA adsorption was fast, reaching ready-state after about 15 min regardless of pH value (Fig. 5; Table 2). The maximum Amide II area estimated by this model reveals a maximum BSA adsorption loading on hematite of 3.70 (arbitrary unit) at pH 5.0, with lower loadings at both lower and higher pH values (Table 2). Achievement of maximum adsorption loading at pH 5 coincides with the pH-isoelectric point of BSA, which is one of the main factors governing the adsorptive properties of proteins^93^.

**Figure 6:**
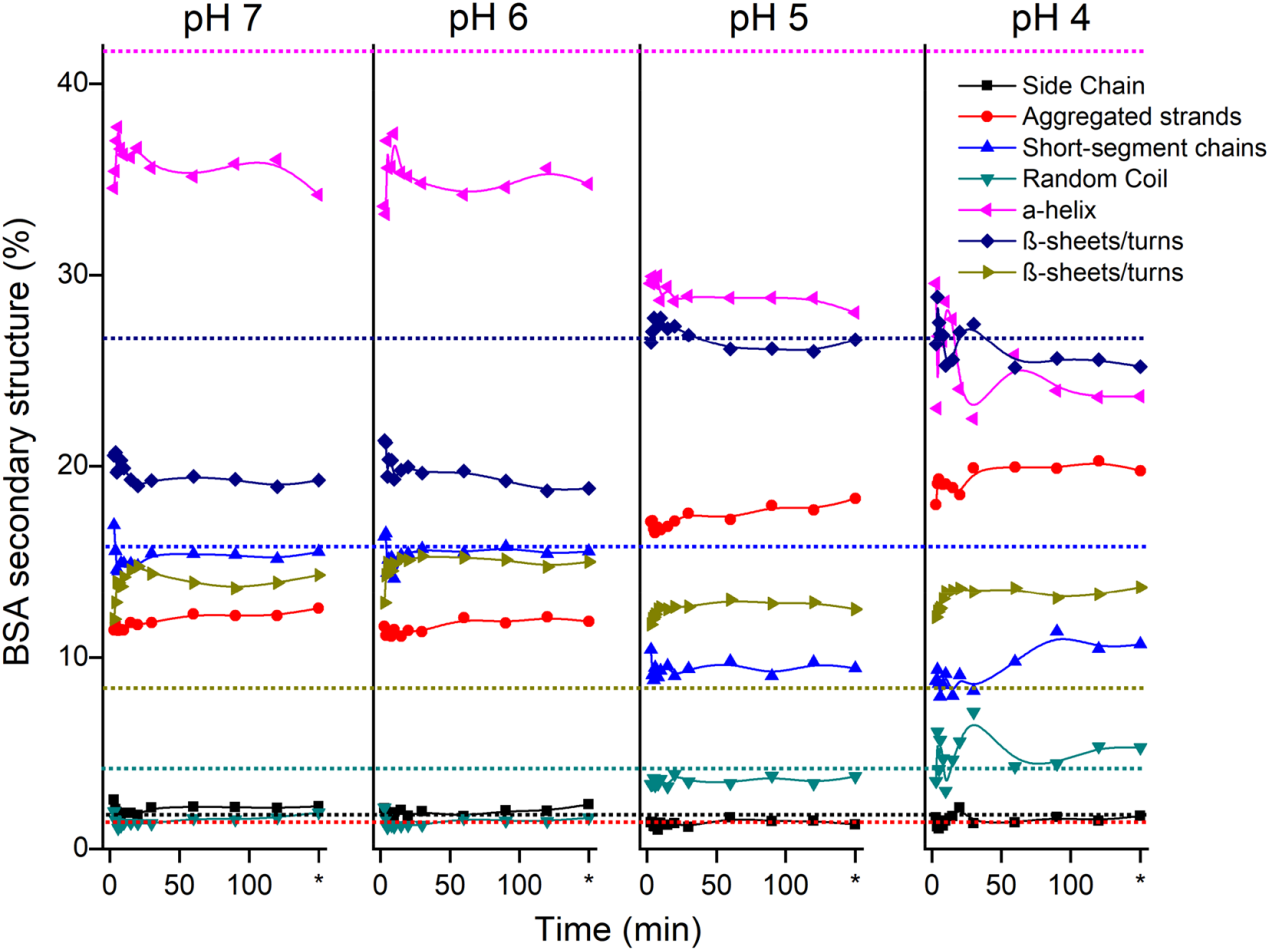
Secondary structure proportion shifts of adsorbed BSA on hematite surface over the kinetic time in different pH values. At 150 min (*) represents the secondary conformation of BSA remained after desorption process. Dotted lines of the corresponding color represent the mean secondary structural composition acquired of free aqueous-BSA in to pH variations. See details in Fig. 2.

Conformational protein analysis as a function of time during BSA adsorption is presented in Fig. 6. The secondary structure of BSA changes as it transfers from the aqueous phase to the hematite surface (Fig. 6), where it undergoes unfolding as indicated by the loss of α-helix and gain of β and extended structures^43,45^. The conformations stabilize around 15 min, which is the same time the overall adsorption process stabilizes (Fig. 5). This indicates that conformational changes are relatively fast.

At pH 7 and 6, there were fast shifts on secondary structure with initial increases of α-helix (1652 cm^–1^) followed by decreases and stabilization; increase at β-sheet/turns (1676 cm^–1^) and decrease of short-segment chains connecting the α-helical segment (1637 cm^–1^). At pH 7, despite electrostatic attraction between the positively charged hematite surface and negatively charged proteins, the repulsive interaction between protein–protein likely limits overall BSA adsorption. At pH 5 and 4, there was increased loss of α-helix (1652 cm^–1^) and subsequent increases in random coil (1644 cm^–1^) and extended chain (1628 cm^–1^) arrangements, this is indicative of more unfolded protein structure. The lowest amount of BSA adsorption however occurs at pH 4 (Fig. 5), where there is electrostatic repulsion not only between proteins (pH ≠ isoeletric point) but also between protein and the hematite surface, which both are positively charged.

Maximum adsorption of BSA occurs at pH 5. We attribute this to: (1) conformational shift that decrease of α-helix (1652 cm^–1^) and short-segment chains (1637 cm^–1^) contents in favor of aggregates strands (1628 cm^–1^) and β-sheet (1662 cm^–1^), as shown in Fig. 6, should lead a increment in total BSA adsorption by dense packaging mechanism^43^, hence (2) the conformational protein changes gradually weakened intermolecular electrostatic repulsion, obeying the multilayer adsorption theory at the gas–liquid interface so that a higher level in protein–protein^109^.

The 2D-COS assessment of the time for the BSA adsorption (Fig. 5SM and Table 2SM) suggest the following sequence of structural changes: pH 7: 1625 cm^–1^ (aggregated strands) → 1612 cm^–1^ (side chain) → 1637 cm^–1^ (short-segment chains) → 1660 cm^–1^ (β-sheet/turns); pH 6: 1685 cm^–1^ (β-sheet/turns) → 1627 cm^–1^ (aggregated strands) → 1640 cm^–1^ (random coil) → 1660 cm^–1^ (β-sheet/turns); pH 5: 1,630 cm^–1^ (aggregated strands) → 1640 cm^–1^ (random coil) → 1656 cm^–1^ (α-helix); and pH 4: 1640 cm^–1^ (random coil) → 1656 cm^–1^ (α-helix). In summary, the initial steps were to expose the hydrophobic core that was previously kept isolated from water. The adsorbed molecule may therefore act as a new adsorption site for a bulk protein or other hydrophobic substances in the system. Hydrophobic forces may therefore be a significant factor in the adsorption of proteins on soil mineral surfaces.

### BSA desorption

BSA desorption was negligible at pH 7 and 4 (Fig. 5) indicating high stability of adsorbed BSA. At pH 5 and 6, however, slight desorption of BSA occurred, amounting to ~ 10% at pH 5 and ~ 5% at pH 6. To characterize desorbed BSA, we calculated the difference spectrum between BSA adsorbed after 120 min and the spectrum acquired after desorption process at pH 5. The result is presented in Fig. 4SM and shows a spectrum resembling that of aqueous BSA at pH 5. This finding suggests that adsorbed BSA complex without shift on secondary structure were preferentially desorbed, which reinforce the protein conformation effects on total protein adsorption. Multilayers formation may lead to low bonding stability for adsorbed protein in the distant layers of the surface, where the effect of mineral surface on protein unfolding becomes lower, following Coulomb's law.

## How much is complex the protein adsorption?

We note several differences between our results and those of earlier studies that used in-situ ATR-FTIR experiments to assess BSA adsorption. Schmidt and Martinez^44^ observed a low absorbance signal and no significant changes in protein secondary structure upon BSA adsorption onto montmorillonite at pH 5, which is quite different than our observations (see Figs. 1SM, 2). The difference in mineral type is probably a key factor. Montmorillonite is a 2:1 aluminosilicate clay with a permanent structural negative charge^110^, while hematite is a variable charge Fe(III)-oxide mineral with a ZPC of 9.3 and thus was positively charged in our experiments. At pH 5, the BSA is slightly negatively charged (isoelectric point = 4.7), which results in electrostatic repulsion with the montmorillonite surface but electrostatic attraction with the hematite surface. Beyond electrostatic effect, the new secondary conformation of BSA allowed optimizes the mineral surface loading.

A very recent study addresses BSA adsorption behavior and conformational change onto hematite with different particle sizes^46^. The authors observed protein unfolding–refolding processes through initial loss of α-helix content after adsorption followed by slow increases at later adsorption times, and point out that unfolded BSA has an open secondary structure enhancing coverage of hematite surface sites that inhibits the adsorption of new molecules. These conclusions reinforce the complexity of studies of protein-mineral.

Our study stressed the pH of equilibrium solution as a paramount variable for kinetics and mechanisms and conformational change of BSA adsorption on hematite. The hematite is positively surface charged (ZPC = 9.3) over the all pH range studied. BSA has a negative charge at pH 7 and 6, which will enhance BSA adsorption by electrostatic attraction. The maximum BSA adsorption occurs at pH 5, near the isoelectric point of BSA, where electrostatic attraction to the surface will be limited. At pH 4, both BSA and mineral surface have a positive charge, causing electrostatic repulsion BSA but the adsorption does occur readily at pH 4. Despite these electrostatic considerations, these results indicate that forces beyond electrostatic interactions between the surface and BSA are involved in the adsorption process such as the electrostatic and steric forces between proteins-protein and protein-mineral, as well as hydrophobic effects from a hydrated layer on the mineral surface.

The pathways by 2D-COS reached an intricate conformational changes involved changes in relatively disordered structures such as aggregated strands and random coil into more compact α-helices form when we assess the pH effect, similar to the effect of BSA concentration described in “BSA adsorption onto hematite: Initial protein concentration effects”. The larger number of cross-peak in synchronous and auto-peaks pathway from pH 7 to pH 4 (Fig. 6) suggests a more complex adsorption process at the high pH value than at low pH.

At high surface coverage, the protein–protein distances decrease and the initial orientation becomes less stable. This may lead to re-orientation involving rotation of the surface-bound proteins, causing perturbation of Amide I^56^ as well as changes in the secondary structure. Since we observe negligible changes in Amide I/Amide II ratio^111,112^ , there is no evidence for protein re-orientation in our BSA-hematite system (Fig. 2SM). IR may be less sensitive to changes protein orientation than other techniques such as Neutron Reflection^56,113^. The potential for re-orientation further emphasizes the complexity of these systems, making trivial hypotheses regarding protein adsorption on mineral surfaces behavior inadvisable^37,51,56,113,114^.

## Conclusions

ATR-FTIR spectroscopic measurements demonstrate that BSA underwent dynamic change of the conformation of adsorbed protein on the hematite surface in response to environmental variables as: initial protein concentration on solution, ionic strength and value of pH medium. These entire variables impacted the total BSA amount adsorbed. The strong dependence of pH stressed the electrostatic force as major effects of total BSA amount adsorbed, which reached the maximum at pH close to isoelectric point to BSA. The isotherm adsorption excluded Langmuir adsorption behavior, suggesting a multilayer BSA adsorption However, the Amide I deconvolution point outed cycle of unfolding or refolding of adsorbed BSA over the environmental variables conditions, in order to optimize the protein conformation for maximize adsorption.

The two-dimensional correlation analysis results showed that BSA adsorption is initially guided by protein-mineral interaction, which represents a first contact of hydrophobic because intense protein unfolding. This step is followed by refolding at higher BSA surface loading, leading the formation of more compact forms of BSA structural arrangement as multilayers/aggregation on mineral surface.

The protein denaturation corresponds to a modification of the secondary and tertiary structure, which was observed here. In the case of enzymes, it is often followed by a loss of activity. The intrinsic properties of proteins and minerals, and diverse scenery of soil solution composition such as pH, ionic strength should be examined to correct estimation of unbounded and immobilized extracellular enzymes. The activities of enzymes adsorbed on soil mineral surfaces require further study.

## Supplementary information

Supplementary information
