# Supplementary material for: The molecular insights into protein adsorption on hematite surface disclosed by in-situ ATR-FTIR/2D-COS study
Source: Sci Rep. 2020 Aug 10;10:13441. doi: 10.1038/s41598-020-70201-z (PMC7417568; doi:10.1038/s41598-020-70201-z)
Supplement: Supplementary file 1 — Supplementary information [file 41598_2020_70201_MOESM1_ESM.doc]

**The molecular insights into protein adsorption on hematite surface disclosed by *in-situ* ATR-FTIR/2D-COS study**

Matheus Sampaio C. Barreto^1, 2*^, Evert J. Elzinga^3^, Luís Reynaldo F. Alleoni^2^

^1^ AgroBiosciences Program, Mohammed VI Polytechnic University (UM6P), Lot 660, Hay Moulay Rachid, 43150, Benguerir, Morocco

^2^ Department of Soil Science, Luiz de Queiroz College of Agriculture (ESALQ), University of São Paulo (USP), Piracicaba, SP, Brazil

^3^ Department of Earth & Environmental Sciences, Rutgers University, Newark, NJ, USA

**SUPPLEMENTARY INFORMATION**


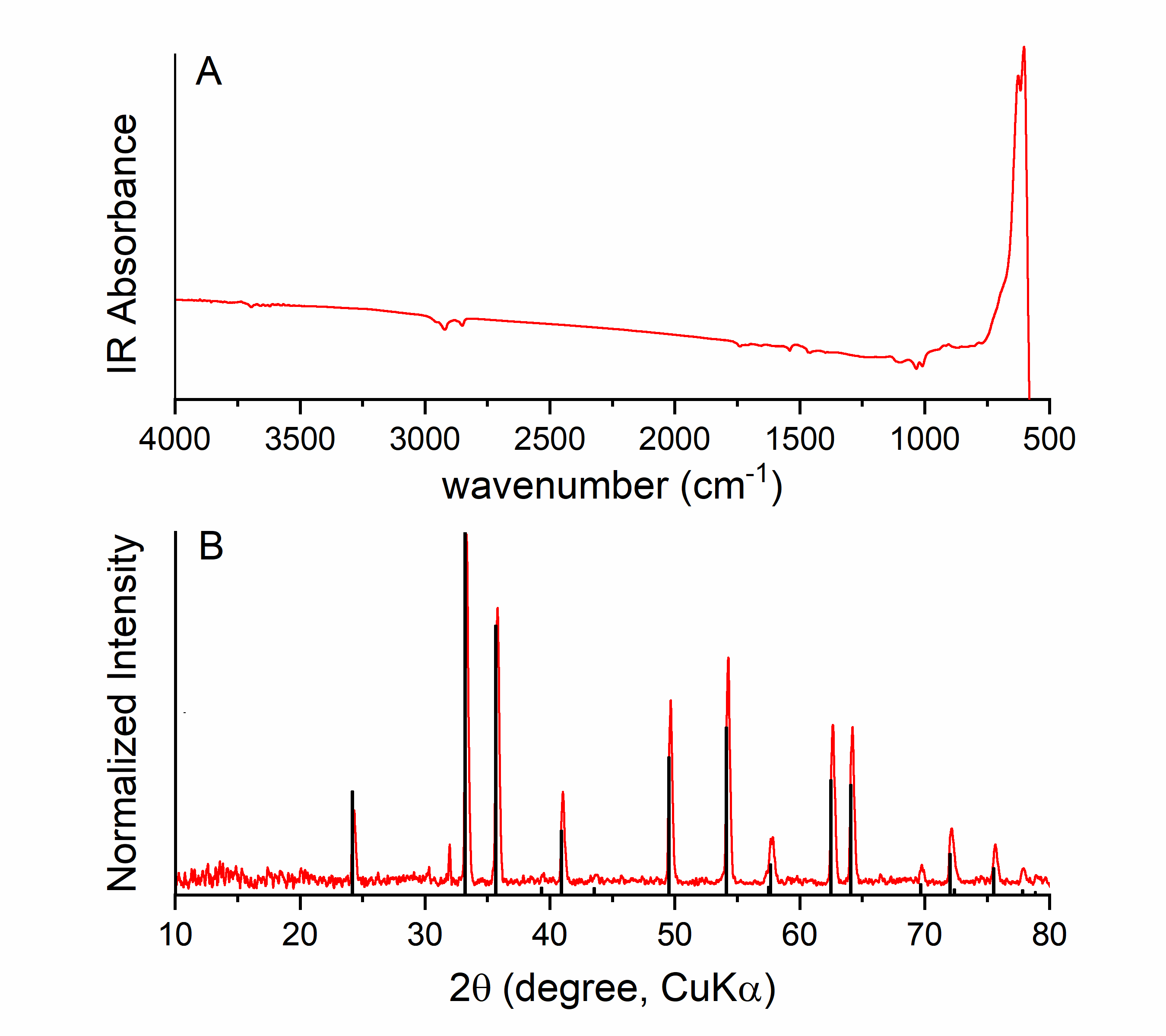


**Figure 1SM. (**A) ATR-FTIR and (B) XRD patterns of hematite used (red lines). Noteworthy that there is no signal of hematite in the regions investigated for BSA adsorption, from 1700 to 1480 cm^-1^. The XRD reference pattern (black bars) available on *American Mineralogist Crystal Structure Database* (AMCSD 0017806). Other crystalline phases were not detected.


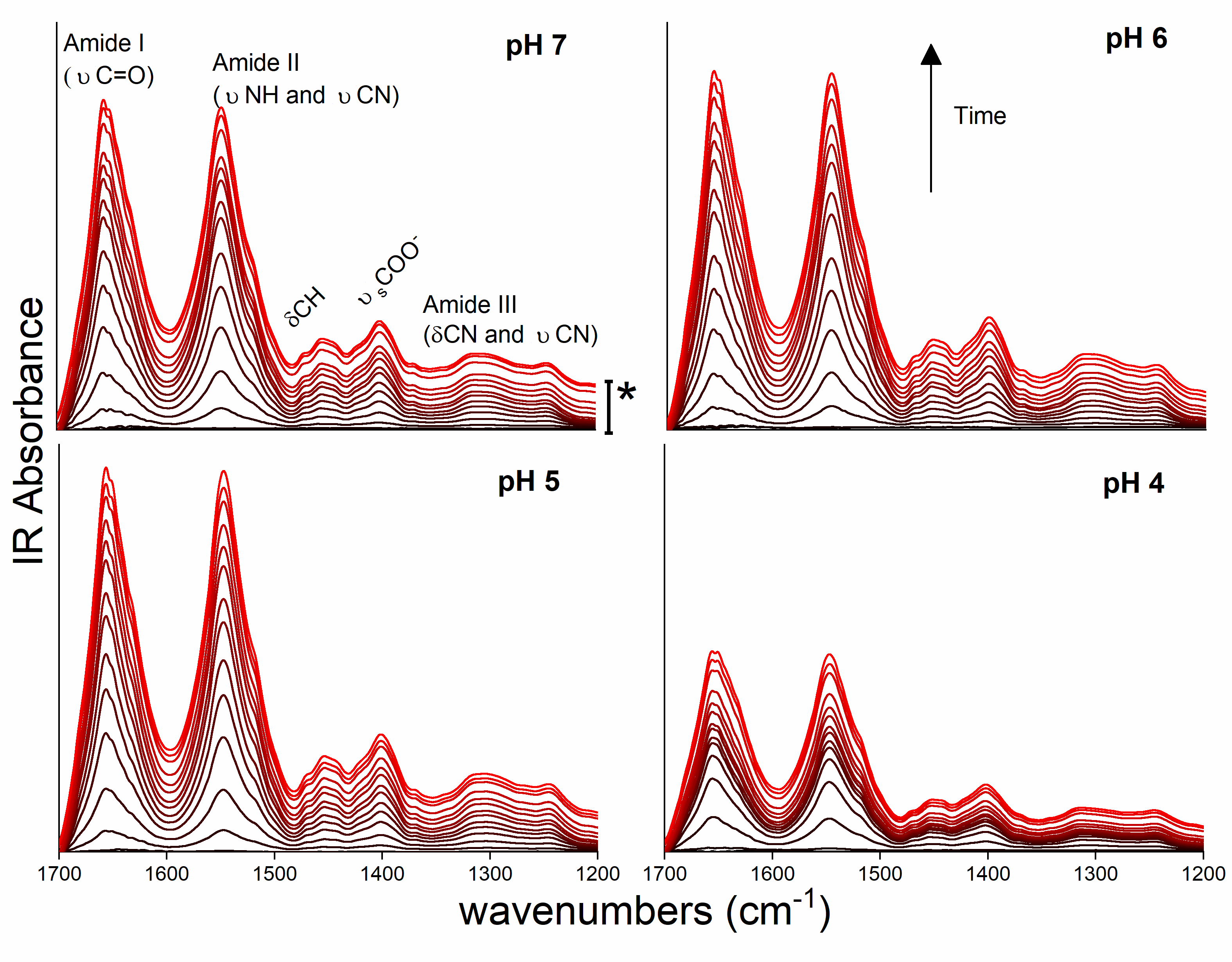


**Figure 2SM.** Raw FTIR spectrum of BSA kinetics adsorption on hematite at pH 7, 6, 5, and 4. The upper-right panel (pH 7) is the same figure presented in Figure 1A in main text. * = instrumental baseline disturb over the adsorption time (120 min). The y-scales of all panels are the same to allow comparison of spectral intensity.


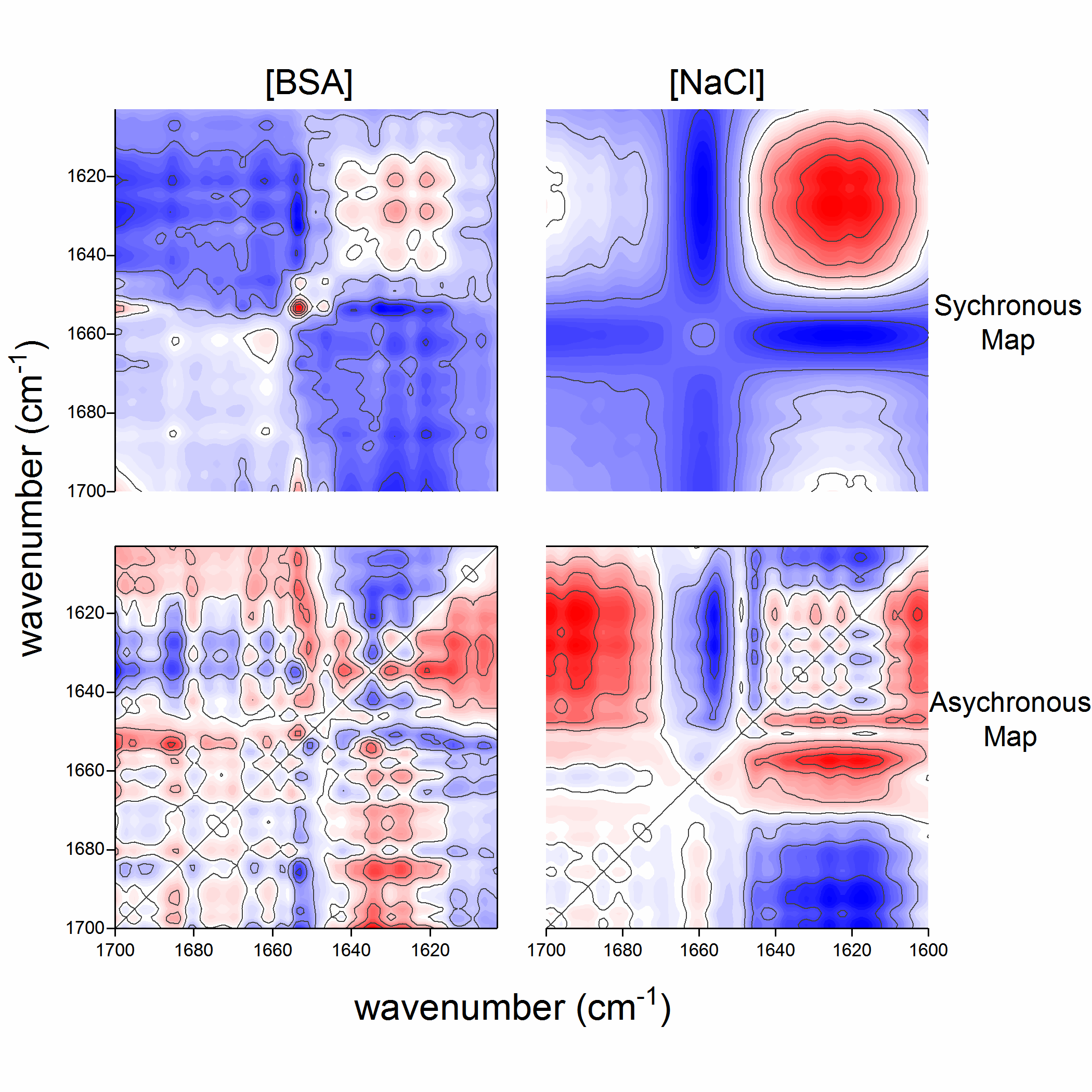


**Figure 3SM.** Synchronous (upper) and asynchronous (bottom) 2D-COS maps generated from the 1700-1600 cm^−1^ (Amide I) regions of FTIR spectra for BSA adsorbed in hematite at over increment of BSA (left) and NaCl (right) concentration. Both experiments were carried out in pH 5. Red and blue represents a positive and negative correlation respectively. The color intensity represents a stronger positive or negative correlation.

**Table 1SM.** Pseudo-second order and Elovich model parameters for BSA adsorption and desorbed fraction for each pH value.

| Pseudo-second order | | | | | | Elovich model | | | | |
| --- | --- | --- | --- | --- | --- | --- | --- | --- | --- | --- |
| pH | AmideII_max_ | | *K* | R^2^ | Reduced χ^2^ | pH | α | β | R^2^ | Reduced χ^2^ |
|  | Peak area (A.U.^2^) | Area peak/Area hematite/min | |  |  |  |  |  |  |  |
| 7 | 1.85 | 0.135 | | 0.82 | 0.20 | 7 | 0.84 | 1.43 | 0.72 | 0.32 |
| 6 | 2.00 | 0.125 | | 0.92 | 0.11 | 6 | 0.92 | 1.20 | 0.83 | 0.23 |
| 5 | 2.05 | 0.131 | | 0.92 | 0.11 | 5 | 1.05 | 1.16 | 0.84 | 0.24 |
| 4 | 1.42 | 0.206 | | 0.95 | 0.01 | 4 | 1.23 | 2.87 | 0.90 | 0.02 |

α represents the initial adsorption rate and β is an empirical constant of activation energy distribution


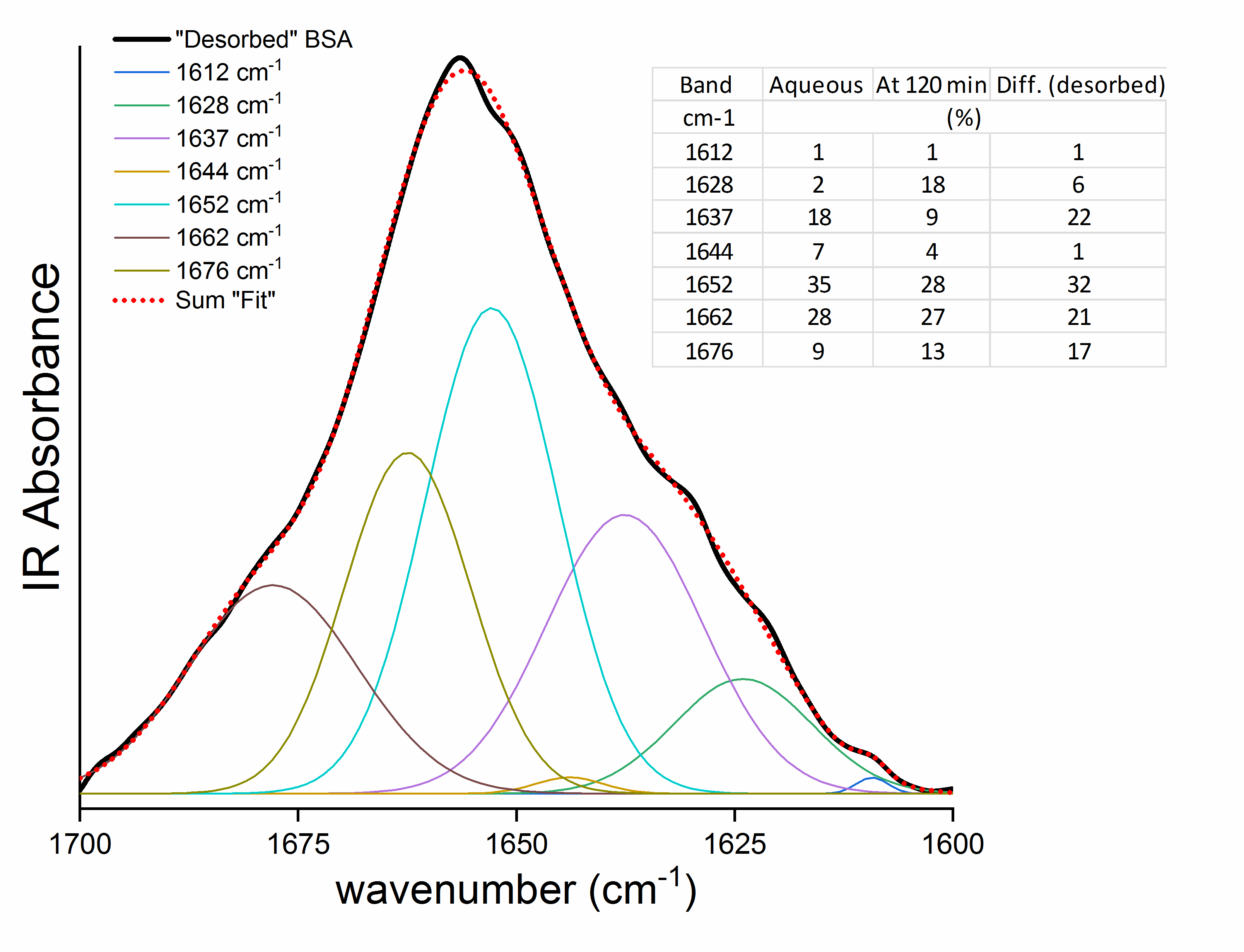


**Figure 4SM.** Deconvolution peak of “desorbed” BSA obtained at pH 5. This spectrum represents the difference between adsorbed spectra after 120 min minus the spectra acquired after 30 min of desorption. The table showed the proportion of each individual Gaussian curves (bands) that composed the raw peaks of aqueous, adsorbed spectra after 120 min and after 30 min of desorption, both realized in pH 5.

**
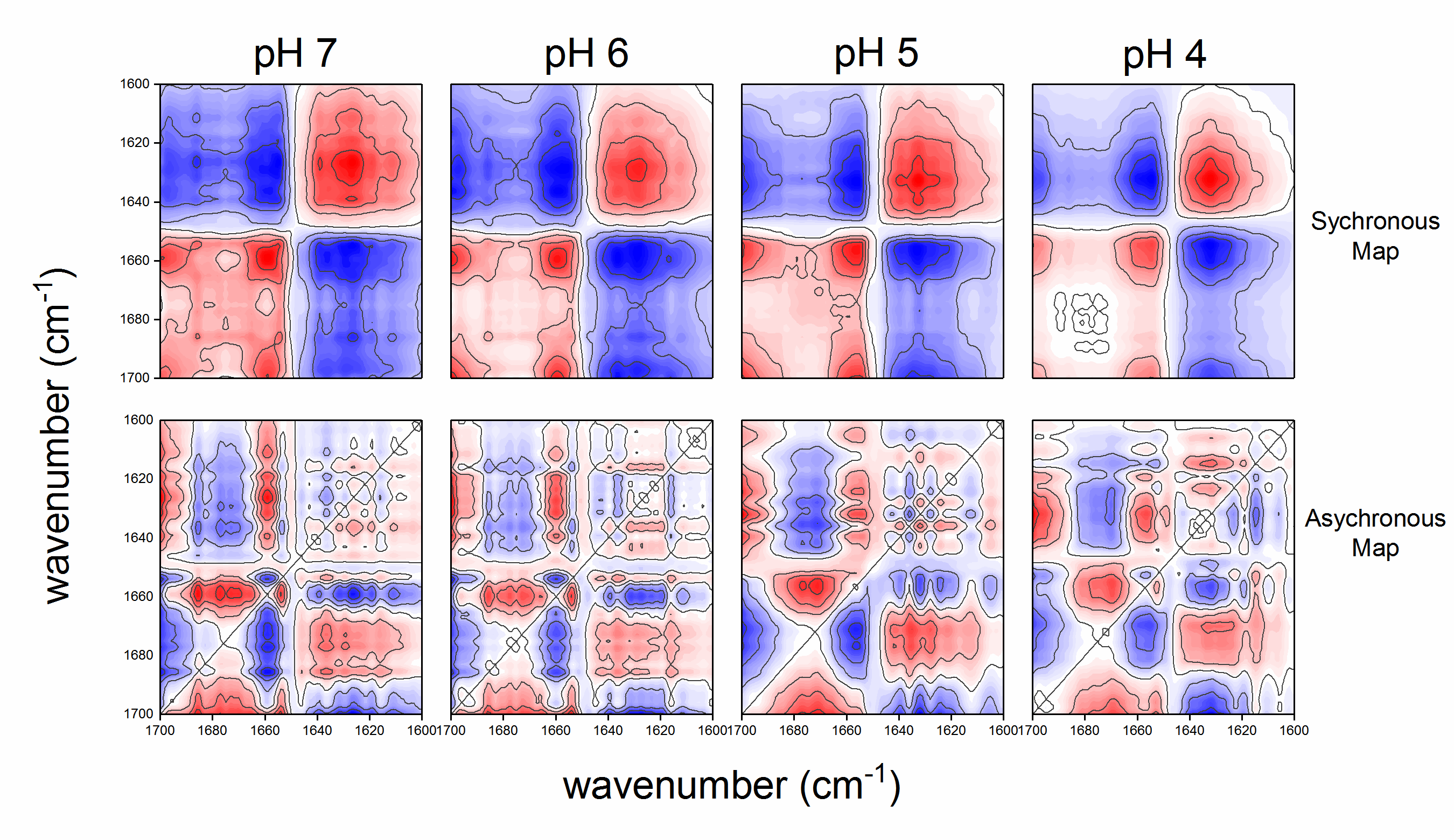
**

**Figure 5SM.** Synchronous (upper) and asynchronous (bottom) 2D-COS maps generated from the 1700-1600 cm^−1^ (Amide I) regions of FTIR spectra for BSA adsorbed in hematite at over time at different pH values. Both experiments were carried out in pH 5. Red and blue represents a positive and negative correlation respectively. The color intensity represents a stronger positive or negative correlation.

**Table 2SM. 2D-COS results of the sign of each cross-peak in synchronous and asynchronous (in parentheses) maps for BSA adsorption on hematite for each perturbation assessment.**

| **BSA perturbation** | | | | | **Process pathway suggested by Noda’ Rules** ^2,3^ | | | | |
| --- | --- | --- | --- | --- | --- | --- | --- | --- | --- |
| BSA concentration | | | | | |  | | | |
|  | 1630 | 1620 |  |  | | |  | 1620→1630→1656 | |
| 1656 | -(+) | -(+) |  |  | | |  |
| 1630 |  | +(-) |  |  | | |  |
|  | | | | | | | | | |
| NaCl concentration | | | | | |  | | | |
|  | 1615 |  |  |  | | |  | 1625→1615 | |
| 1625 | +(+) |  |  |  | | |  |
| * Weak peaks in asynchronous map, see figure 3SM | | | | | | | | | |
| Adsorption kinetic (see figure 8) | | | | | | | | | |
| pH 7 | | | | | | | | | |
|  | 1660 | 1640 | 1625 | 1612 | | |  |  |  |
| 1685 | +? | -(-) | -(-) | -(-) | | |  | 1625→1612→1640  →1660 | |
| 1660 |  | -(+) | -(+) | -(+) | | |  |
| 1640 |  |  | +(-) | +(-) | | |  |
| 1625 |  |  |  | +(+) | | |  |
| pH 6 | | | | | |  | | | |
|  | 1660 | 1640 | 1627 |  | | |  |  |  |
| 1685 | +(+) | -(-) | -(-) |  | | |  | 1685→1627→1640  →1660 | |
| 1660 |  | -(+) | -(+) |  | | |  |
| 1640 |  |  | +(-) |  | | |  |
|  |  |  |  |  | | |  |
| pH 5 | | | | | |  | | | |
|  | 1640 | 1630 |  |  | | |  |  |  |
| 1656 | -(+) | -(+) |  |  | | |  | 1630→1640→1656 | |
| 1640 |  | +(-) |  |  | | |  |
| pH 4 | | | | | |  | | | |
|  | 1640 |  |  |  | | |  | 1640→1656 | |
| 1656 | -(+) |  |  |  | | |  |

Signs were obtained in the upper-left corner of the maps: +, positive; −, negative. Only the strongest cross-peak in synchronous and auto-peaks in synchronous map (peaks in diagonal line) were considerate appropriate to “reaction sequence” assessment.

**REFERENCES**

1. Yang, H., Yang, S., Kong, J., Dong, A. & Yu, S. Obtaining information about protein secondary structures in aqueous solution using Fourier transform IR spectroscopy. *Nat. Protoc.* **10**, 382–396 (2015).

2. Noda, I. & Ozaki, Y. Principle of Two-Dimensional Correlation Spectroscopy. in *Two-Dimensional Correlation Spectroscopy - Applications in Vibrational and Optical Spectroscopy* 15–38 (John Wiley & Sons, Ltd, 2005). doi:10.1002/0470012404.ch2

3. Noda, I. Vibrational two-dimensional correlation spectroscopy (2DCOS) study of proteins. *Spectrochim. Acta Part A Mol. Biomol. Spectrosc.* **187**, 119–129 (2017).
